# Antitumor Effect of KML-B-Treated Dendritic Cells via Induction of Lymphocyte Activation

**DOI:** 10.1155/2017/2471627

**Published:** 2017-05-29

**Authors:** Jong-Jin Kim, Yun-Ho Hwang, Kyung-Yun Kang, Sung-Ju Lee, Jong-Bae Kim, Jina Choi, Sung-Tae Yee

**Affiliations:** ^1^Singapore Bioimaging Consortium, Agency for Science, Technology and Research, 11 Biopolis Way, No. 02-02 Helios, Singapore 138667; ^2^Department of Pharmacy, Sunchon National University, 255 Jungangno, Suncheon 540-950, Republic of Korea; ^3^Mistle Biotech Co. Ltd, Pohang 37668, Republic of Korea; ^4^Department of Hematology-Oncology, Chonnam National University Hwasun Hospital, Hwasun 519-763, Republic of Korea; ^5^Suncheon Research Center for Natural Medicines, Suncheon, Republic of Korea

## Abstract

Lectins are carbohydrate-binding proteins with various biological activities, such as antitumor and immunomodulatory effects. Although lectins have various biological activities, they are still limited by cytotoxicity in normal cells. To overcome this problem, we used the noncytotoxic part of Korean mistletoe lectin B-chain (KML-B) to induce maturation of dendritic cells (DCs). A previous study reported that KML-B induces DC maturation by triggering TLR-4, including expression of costimulatory molecules (CD40, CD80, and CD86), MHC II, and secretion of cytokines in DCs. Additionally, matured DCs by KML-B induced T helper (Th) cell activation and differentiation toward Th1 cells. However, the interaction of KML-B-treated DCs with CD8^+^ T cells is still poorly understood. In this study, we confirmed the ability of matured DCs by KML-B to stimulate cytotoxic T cells using OT-1 mouse-derived CD8^+^ T cells. KML-B induced MHC I expression in DCs, stimulation of CD8^+^ T cell activation and proliferation, and IFN-*γ* secretion. Moreover, tumor sizes were reduced by KML-B treatment during vaccination of OVA_257−264_-pulsed DCs. Here, we confirmed induction of CD8^+^ T cell activation and the antitumor effect of KML-B treatment in DCs.

## 1. Introduction

To overcome the generation and aggravation of cancer, many treatment methodologies have been developed, including drugs, surgeries, and chemo- and radiotherapy. Despite these treatments, current treatment approaches have clinical limitations [1]. The immune system of many cancer patients does not appropriately respond to cancer cells [2]. Inefficient antigen presentation to helper and cytotoxic T cells is responsible for inhibition of the anticancer immune response by antigen-presenting cells (APCs) [3]. In tumors, antigen-presenting function and induction of the immune response are inhibited due to the inhibitory activities of tumor cells and the presence of tumor-derived TGF-*β*, regulatory T cells (Treg), tumor-associated macrophages, tumor-associated neutrophils, and myeloid-derived suppressor cells (MDSCs) [4].

To evade immunosuppression during cancer in vivo, antigen-specific activated immune cells are transplanted to the body after activation in vitro for immunotherapy. Dendritic cells (DCs) are the most promising candidate for immunotherapy since they initiate the adaptive immune response. In addition, DCs stimulate both helper and cytotoxic T cells (CD4^+^ cells and CD8^+^ T cells) by antigen cross-presentation [5]. TLR ligands are promising immunoadjuvants for immunotherapy since they stimulate many kinds of immune cells and initiate activation of antigen presentation by APCs [6]. A previous study reported that KML-B (B chain of Korean mistletoe lectin) induces maturation of DCs by triggering toll-like receptor-4 (TLR-4) signaling [7]. Induction of the Th1-type immune and cytotoxic T lymphocyte (CTL) responses is necessary for effective anticancer immunotherapeutic strategies for cancer. In particular, TLR-4 is known to induce the Th1 response, and its ligands are candidate immunostimulatory adjuvants for cancer therapy [8].

Many lectins have been examined as immunoadjuvant candidates in biological and therapeutic research studies, as they have been shown to interact with glycan-linked receptors on cell surfaces to prime cell signaling and biological responses [9–13]. Korean mistletoe (*Viscum album coloratum*) lectin was reported to have various biological activities. However, although KML has various biological and immunological activities, its use is limited in cancer therapy or as an adjuvant due to its toxicity in normal cells [14]. A previous study confirmed that the nontoxic part of KML-B exhibits potent immunomodulatory properties via induction of DC maturation, which increases the Th1-type immune response and decreases Treg cell activation, suggesting it can be considered a potential DC-based cancer therapy and immunoadjuvant [7].

In this study, we confirmed activation of CD8^+^ T cells and its antitumor activity by treatment with KML-B-treated DCs.

## 2. Materials and Methods

### 2.1. Animals

Female C57BL/6 mice (7 to 8 weeks old) were bred and maintained under specific pathogen-free conditions at Dae Han Bio Link (Eumseong, Korea). C57BL/6 OT-1 T cell receptor (TCR) transgenic mice were obtained from Jackson Laboratories. All mice were treated in strict accordance with the guidelines issued by the Sunchon National University Institutional Animal Care and Use Committee (SCNU_IACUC-2013-4) for the care and use of laboratory animals.

### 2.2. Reagents and Antibodies

Recombinant mouse granulocyte-macrophage colony-stimulating factor (GM-CSF) and interleukin- (rmIL-) 4 were purchased from R&D Systems. Mitomycin C (MMC) was purchased from Sigma-Aldrich. Carboxyfluorescein succinimidyl ester (CFSE) and LPS were purchased from Invitrogen. OVA peptide (OVA_257−264_) was purchased from InvivoGen. The following FITC-, PE-, or APC-conjugated monoclonal antibodies (Abs) and nonlabeled Abs were purchased from BD Biosciences: CD8 (53-6.7), CD16/32 (2.4G2), CD11c (HL3), H-2kb (AF6–88.5), and IFN-*γ* (XMG1.2). Cytokine ELISA primary and secondary antibodies for murine IL-4 and IFN-*γ* were purchased from BD Biosciences.

### 2.3. Preparation of Korean Mistletoe Lectin B-Chain (KML-B)

KML-B was prepared as previously described [15, 16]. Briefly, subchains of KML were dissociated with 5% *β*-mercaptoethanol in PBS for 16 hours at 25°C. The solution was then loaded onto a lactose affinity column (Sigma-Aldrich), and unbound materials were washed out with equivalent buffer. Bound B-chain was eluted using 0.1 M lactose in PBS and dialyzed with PBS. Protein concentrations were determined using a BCA protein assay kit, and KML-B was stored in PBS at −80°C until required.

### 2.4. Generation of BMDCs

BMDCs were derived from C57BL/6 mouse bone marrow cells. Briefly, cells from bone marrow flushed from femurs and tibiae of mice were cultured in 6-well tissue culture plates at 1 × 10^6^ cells/ml in complete RPMI culture medium supplemented with 10% FBS and 2-mercaptoethanol (50 *μ*M/ml) in the presence of IL-4 (1000 U/ml) and GM-CSF (1000 U/ml). On days 2 and 4, fresh medium and cytokines were added after removing nonadherent cells. The immature BMDCs obtained on day 6 were used in subsequent experiments.

### 2.5. Flow Cytometry

BMDCs (1 × 10^6^ cells) were incubated for 18 hours with or without various concentrations of KML-B or LPS (1 *μ*g/ml). BMDCs were then harvested and washed with flow cytometry buffer. Fc*γ*II and Fc*γ*III receptors on BMDCs were blocked by incubation with anti-mouse CD16/32 (1 *μ*g/1 × 10^6^ cells) for 30 min on ice. Cells were then stained with fluorescence-labeled antibodies (1 *μ*g/1 × 10^6^ cells) specific for the following markers: anti-mouse CD11c-FITC and MHC I-PE. Following 30 min of incubation on ice, cells were washed with flow cytometry buffer and read on a BD FACScanto™ II. Data analysis was performed using BD FACS Diva software or FlowJo program.

### 2.6. CD8^+^ OT-1 Cell Proliferation

BMDCs were incubated for 18 hours with or without LPS (1 *μ*g/ml), KML-B, or OVA_257−264_. Matured BMDCs were harvested and then treated with MMC (50 *μ*g/ml, Sigma-Aldrich). Splenocytes were isolated from C57BL/6 OT-1 T cell receptor (TCR) transgenic mice. Splenocytes were then washed in PBS and labeled with 1 *μ*M CFSE in PBS. After CFSE labeling, splenocytes (1 × 10^5^) were cocultured with, in various conditions, treated BMDCs (1 × 10^4^) in 96-well U-bottom plates. After 2 or 3 days, cells were harvested and washed in PBS. The proliferation of OVA_257−264_-specific CD8^+^ T cells was evaluated by flow cytometry after staining with anti-mouse CD8-APC.

### 2.7. Intercellular Cytokine Staining

BMDCs were incubated for 18 hours with or without LPS (1 *μ*g/ml), KML-B, or OVA_257−264_. Matured BMDCs were harvested and then treated with MMC (50 *μ*g/ml, Sigma-Aldrich). Splenocytes were isolated from C57BL/6 OT-1 T cell receptor (TCR) transgenic mice. Splenocytes (1 × 10^5^) were cocultured with, in various conditions, BMDCs (1 × 10^4^) in 96-well U-bottom plates. After 48 hours, splenocytes were assayed for intracellular cytokines by flow cytometry. CD8^+^ T cells were stained with anti-CD8-PE. Cells were fixed and permeabilized with 4% Fixation/Perm buffer III (BD Biosciences) and stained with anti-IFN-*γ*-FITC. Finally, CD8^+^ T cells were gated and analyzed on a BD FACScanto II.

### 2.8. Cytokine Assay

Levels of various cytokines were measured in cell culture supernatants. Cytokine levels were measured by ELISA. The lower detection limits of these assays were 1.11 pg/ml for IL-4 and IFN-*γ*. All samples were tested in triplicate for standard curves.

### 2.9. Antitumor Activity

C57BL/6 mice were subjected to subcutaneous injection of 1 × 10^5^ E.G7 cells, an OVA-expressing EL4 variant, into a flank site and then injected intravenously with PBS, untreated DCs (imDC), DCs pulsed with OVA_257−264_ (OVA-DC), or KML-B (500 ng/ml)-treated DCs pulsed with OVA_257−264_ (OVA-KML-B-DC) on days 0, 2, and 4 after tumor inoculation. Tumor growth was monitored and measured every 2 days (*n* = 8 mice/group).

### 2.10. Statistical Analysis

Results are presented as means ± SDs. Statistically significant differences between groups were identified by one-way analysis of variance (ANOVA) using SPSS version 22 (Chicago, IL) with Duncan's multiple-range test. Values were considered to be statistically significant when *p* < 0.05.

## 3. Results

### 3.1. KML-B Induces Expression of MHC I Molecules on BMDCs

**Figure 1:**
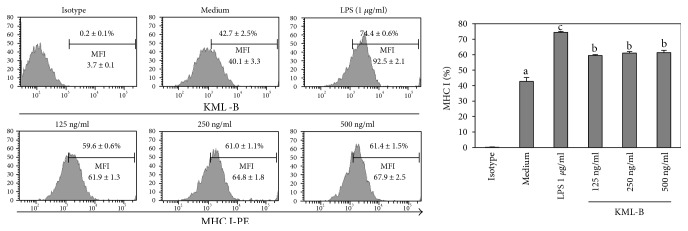
KML-B-induced expression of MHC class I in BMDCs. BMDCs were treated with the indicated concentrations of LPS or KML-B for 18 hours. Flow cytometry was used to analyze expression levels of costimulatory molecules on CD11c^+^-gated BMDCs. Results are representative of three experiments. ^a,b,c^The means not sharing a common letter are significantly different among groups at *p* < 0.05 by Ducan's multiple-range test.

A previous study reported that KML-B induces phenotypic and functional maturation of BMDCs; also, we confirmed that those phenomena were not affected by LPS [7]. In this experiment, we tested whether or not KML-B induces expression of major histocompatibility complex class I (MHC I) on BMDCs. LPS served as a positive control. Untreated BMDCs expressed basal levels of MHC I (42.7 ± 2.5%) (Figure 1). As expected, expression of MHC I was higher on BMDCs treated with LPS (74.4 ± 0.6%). Likewise, the treatment of BMDCs with KML-B (500 ng/ml) significantly increased expression of MHC I (61.4 ± 1.5%). In addition, as indicated in Figure 1, medium intensity of fluorescence (MFI) was increased with similar pattern with percent. These findings show that MHC I expression was upregulated in KML-B-treated BMDCs, similar to the level of LPS-treated BMDCs.

### 3.2. KML-Treated BMDCs Induce OT-1 T Cell Activation

**Figure 2:**
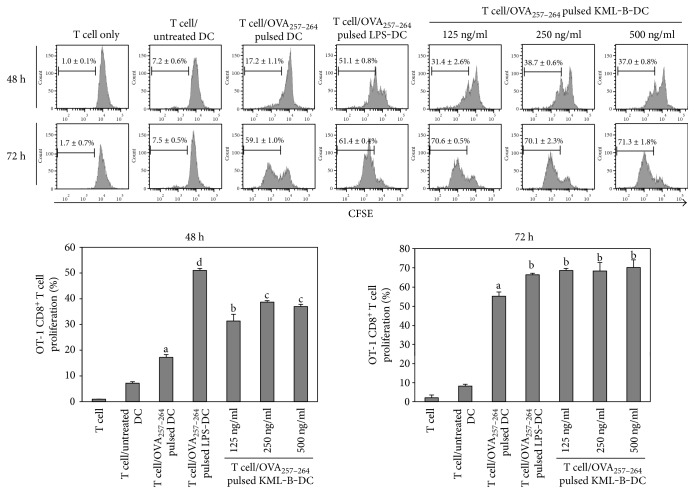
KML-B-treated BMDCs induced proliferation of OT-1 mouse-derived CD8^+^ T cells. Splenocytes from the OT-1 mouse were stained with CSFE and cocultured for 48 or 72 hours with BMDCs which was treated with LPS or KML-B and pulsed with OVA_257−264_ peptide (1 *μ*g/ml). Proliferation of OVA_257−264_ peptide-specific CD8^+^ T cells was assessed by flow cytometry after staining by anti-mouse CD8-APC. Results are representative of three experiments. ^a,b,c,d^The means not sharing a common letter are significantly different among groups at *p* < 0.05 by Ducan's multiple-range test.

As mentioned above, expression of MHC I was upregulated on BMDCs by KML-B treatment. MHC I is an important molecule in the activation of CD8^+^ T cells. For this reason, CD8^+^ T cell activation by KML-B-treated BMDCs was investigated using splenocytes from OT-1 mice. Proliferation and cytokine production of CD8^+^ T cells were measured by anti-CD8-APC staining after splenocytes from OT-1 mice were cocultured with various BMDCs for 48 or 72 hours. CD8^+^ T cells cocultured with KML-B-treated BMDCs pulsed with OVA_257−264_ showed greater proliferation than those cocultured with untreated BMDCs after 48 hours. In addition, proliferation of CD8^+^ T cells which was cocultured with KML-B-treated BMDCs pulsed with OVA_257−264_ was more increased in the 72-hour incubation time than in the 48-hour incubation time (Figure 2), demonstrating that KML-B acted as a potent immunostimulator of CD8^+^ T cells via DC activation.

**Figure 3:**
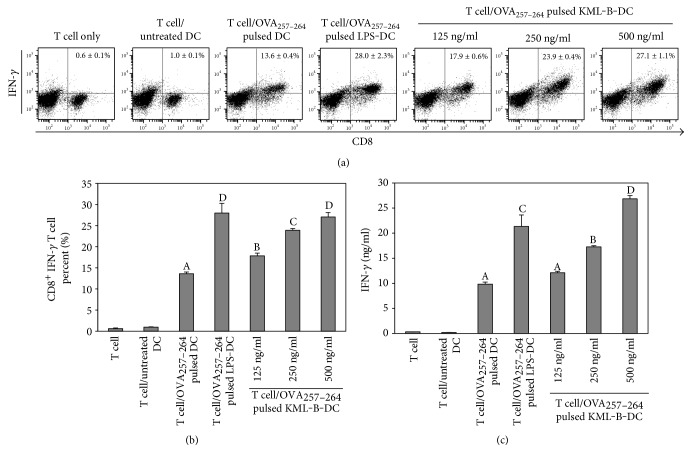
KML-B-treated BMDCs induced IFN-*γ* secretion of OT-1 mouse-derived CD8^+^ T cells. Splenocytes from the OT-1 mouse were cocultured for 48 hours with BMDCs which was treated with LPS or KML-B and pulsed with OVA_257−264_ peptide (1 *μ*g/ml). IFN-*γ* production was measured by intercellular staining and ELISA in cells and supernatants, respectively. Results are representative of three experiments. ^A,B,C,D^The means not sharing a common letter are significantly different among groups at *p* < 0.05 by Ducan's multiple-range test.

We then investigated IFN-*γ* production in CD8^+^ T cells activated with KML-B-treated BMDCs pulsed with OVA_257−264_ for 48 hours. CD8^+^ T cells primed with KML-B-treated BMDCs produced significantly higher levels of IFN-*γ* than cells primed with untreated BMDCs (Figures 3(a) and 3(b)). ELISA studies also revealed high levels of IFN-*γ* in the supernatant of KML-B-treated BMDCs pulsed with OVA_257−264_ (Figure 3(c)). These results provide evidence that KML-B acted as an efficient immunostimulator of CD8^+^ T cells via induction of DC activation.

### 3.3. KML-B-Treated DCs Enhance Antitumor Efficacy

**Figure 4:**
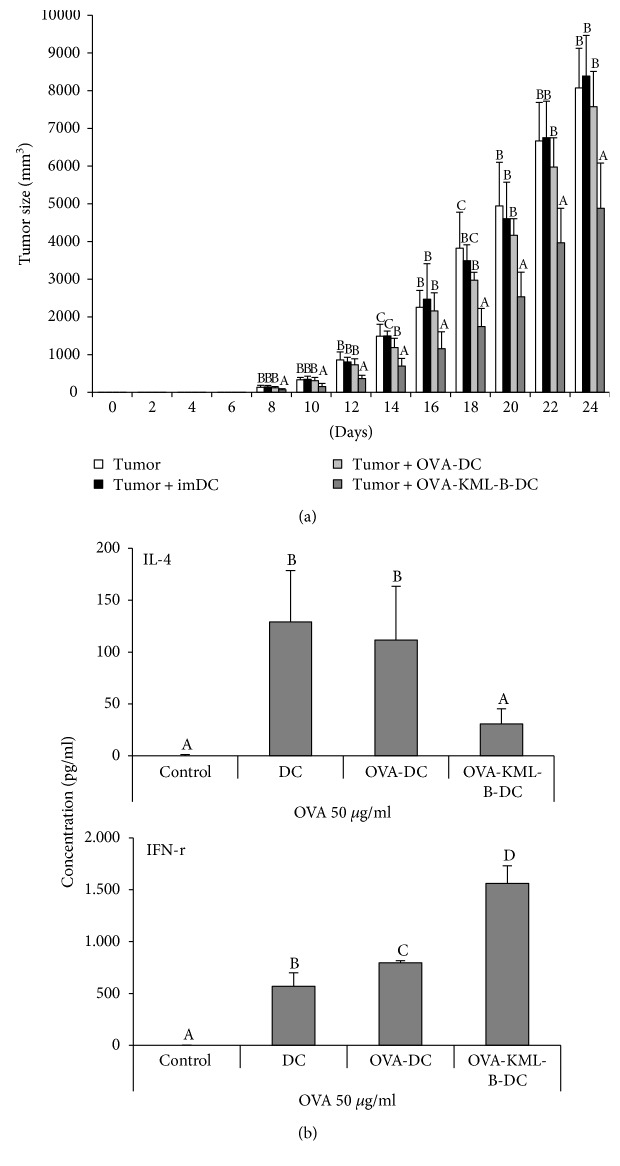
KML-B-treated BMDCs suppressed the E.G7 tumor size in mouse models. C57BL/6 mice were injected subcutaneously (flank site) with 1 × 10^5^ E.G7 cells and then injected intravenously (tail vein) with PBS, untreated DCs (imDC), DCs pulsed with OVA_257−264_ (OVA-DC), or KML-B (500 ng/ml)-treated DCs pulsed with OVA_257−264_ (OVA-KML-B-DC) on days 0, 2, and 4 after tumor challenge ((a), *n* = 8 mice/group). ^A,B,C^The means not sharing a common letter are significantly different among groups at *p* < 0.05 by Ducan's multiple-range test. (b) E.G7 tumor-inoculated mice were injected intravenously with PBS, untreated DCs (imDC), DCs pulsed with OVA_257−264_ (OVA-DC), or KML-B (500 ng/ml)-treated DCs pulsed with OVA_257−264_ (OVA-KML-B-DC) on days 0 and 7. On day 14, splenocytes were harvested and treated with OVA_257−264_ (1 *μ*g/ml) for 24 hours (*n* = 3 mice/group). Cytokines were measured from the supernatant. ^A,B,C,D^The means not sharing a common letter are significantly different among groups at *p* < 0.05 by Ducan's multiple-range test.

In the in vitro experiment, KML-B treatment induced MHC class I expression on DCs, and this phenomenon subsequently induced CD8^+^ T cell activation. Therefore, to confirm whether or not KML-B-treated DCs upregulate antitumor activity in vivo, mice were injected with PBS, untreated DCs (imDC), DCs pulsed with OVA_257−264_ (OVA-DC), or KML-B-treated DCs pulsed with OVA_257−264_ (OVA-KML-B-DC) on days 0, 2, and 4 after tumor inoculation and then monitored for tumor growth for 24 days. As shown in Figure 4(a), KML-B-treated DCs significantly suppressed E.G7 tumor growth compared to that of tumors in mice that received imDCs and OVA-DC. After vaccination of each DC type into normal mice, we checked cytokine secretion in splenocytes to determine what types of immunity increased.  Figure 4(b) indicates that IFN-*γ* secretion was upregulated in the OVA-KML-B-DC-vaccinated group compared to the OVA-DC group. Interestingly, IL-4 secretion was reduced in the OVA-KML-B-DC group. These data mean that cellular immune responses were enhanced by KML-B treatment to DCs.

## 4. Discussion

Our immune systems are unable to respond to many kinds of tumors due to the ability of many cancer cells to evade host immunity [17]. In an attempt to boost immune functions for detection of low immunogenicity tumors, we previously reported that KML-B, a nontoxic subchain from KML, induces DC maturation in the form of surface molecule expression (CD40, CD80, and CD86), MHC class II, cytokine secretion (IL-1*β*, IL-6, IL-12p70, and TNF-*α*), and antigen presentation function to CD4^+^ T cells [7]. Finally, these responses are characteristic of Th1 cell immunity in vitro and in vivo. The Th1 cell immune response is very important to establishing cellular immunity against pathogens and cancer [18]. Moreover, to overcome tumor growth, CTL activity is important since CTLs directly kill tumor cells [19].

In this study, we determined whether or not KML-B-treated DCs can activate the CD8^+^ T cell response. DCs are able to present exogenous antigens to MHC class I molecules by cross-presentation [5]. KML-B treatment induced expression of MHC class I on DCs. To activate CD8^+^ T cells, several signals such as MHC class I, costimulatory molecules (CD80 and CD86), and cytokines are needed [5]. We already confirmed that KML-B enhances secretion of IL-12 and expression of costimulatory molecules by DCs. In addition, matured DCs by KML-B-induced secretion of IFN-*γ* from CD4^+^ T cells. Synthetically, these phenomena suggest that DCs matured by KML-B promote CD8^+^ T cell activation. As shown above, KML-B-treated OVA_256−264_-pulsed DCs incubated with peptide-specific CD8^+^ T cells from OT-1 mice enhanced proliferation of CD8^+^ T cells compared with OVA_256−264_-pulsed DCs treated to CD8^+^ T cells. For the other activation markers, production and secretion of IFN-*γ* were also elevated from CD8^+^ T cells cocultured with KML-B-treated DCs. IFN-*γ* is an important cytokine in the anticancer response, as it induces macrophage activation, increases MHC molecule expression, and enhances the Th1 cell immune response [20]. To sum up, KML-B-treated DCs clearly induce CD8^+^ T cell activation, including proliferation and cytokine secretion.

As KML-B was shown to be a potential immunoadjuvant in DC vaccination, we next applied KML-B-treated DCs in an in vivo tumor model to prevent tumor growth.  Figure 4 indicates that tumor growth was significantly reduced by OVA-pulsed KML-B-treated DCs. In addition, the secretion pattern of cytokines also changed by KML-B. IL-4 is an inhibitory cytokine of the cellular immune response and secreted from a Th2 cell. As reported in the previous study, this study also confirmed an inhibitory effect of the Th2 response [7]. Furthermore, IFN-*γ* which secreted from activated Th1 and CD8^+^ T cells is increased by KML-B-treated DCs. We suggest that this phenomenon was induced by activation of lymphocytes such as Th1 cells and CD8^+^ T cells, and this explanation is supported by cytokine (IL-4 and IFN-*γ*) production in splenocytes from OVA-KML-B-DC-immunized mice. Finally, these kinds of responses inhibited tumor growth compared with those of the other groups in vivo.

## 5. Conclusion

Taken together, we confirmed the ability of KML-B-treated DCs to induce CD8^+^ T cell activation, which enhanced the anticancer response in an in vivo E.G7 tumor mouse model. Moreover, a previous study reported that KML-B-treated DCs enhance the Th1 immune response. Thus, we suggest that the nontoxic part of KML-B can be a potent immunoadjuvant for vaccination. In addition, KML-B-treated DCs can be used as a DC-based immunotherapy for tumor treatment.
